# ^1^H NMR-Based Metabolite Profiling of Planktonic and Biofilm Cells in *Acinetobacter baumannii* 1656-2

**DOI:** 10.1371/journal.pone.0057730

**Published:** 2013-03-06

**Authors:** Jinki Yeom, Ji-Hyun Shin, Ji-Young Yang, Jungmin Kim, Geum-Sook Hwang

**Affiliations:** 1 Integrated Metabolomics Research Group, Seoul Center, Korea Basic Science Institute, Seoul, Republic of Korea; 2 Department of Microbiology, Kyungpook National University School of Medicine, Daegu, Republic of Korea; 3 Department of Pharmacology, Dankook University College of Medicine, Cheonan, Republic of Korea; 4 Graduate School of Analytical Science and Technology, Chungnam National University, Daejeon, Republic of Korea; Imperial College London, United Kingdom

## Abstract

*Acinetobacter baumannii* is an aerobic and gram-negative pathogenic bacterium that is resistant to most antibiotics. Recently, *A. baumannii* 1656-2 exhibited the ability to form biofilms under clinical conditions. In this study, global metabolite profiling of both planktonic and biofilm forms of *A. baumannii* 1656-2 was performed using high-resolution nuclear magnetic resonance (NMR) spectroscopy and multivariate statistical analysis to investigate the metabolic patterns leading to biofilm formation. Principal components analysis (PCA) and orthogonal partial least-squares discriminant analysis (OPLS-DA) score plots showed a distinct separation between planktonic and biofilm cells. Metabolites including acetates, pyruvate, succinate, UDP-glucose, AMP, glutamate, and lysine were increasingly involved in the energy metabolism of biofilm formation. In particular, the ratio of *N-*acetyl-D-glucosamine (GlcNAc) to D-glucosamine (GlcNH_2_) was significantly higher during biofilm formation than under the planktonic condition. This study demonstrates that NMR-based global metabolite profiling of bacterial cells can provide valuable insight into the metabolic changes in multidrug resistant and biofilm-forming bacteria such as *A. baumannii* 1656-2.

## Introduction

*Acinetobacter* species have been isolated from a variety of habitats, including human clinical specimens, water and soils, attesting the profound adaptability of the genus to various environments [1]–[3]. Studies on *Acinetobacter* species have focused primarily on *Acinetobacter baumannii* due to the clinical importance of its multidrug-resistant (MDR) strains [2], [3]. *A. baumannii* is an aerobic Gram-negative coccobacillus that causes nosocomial human infections, particularly in immunocompromized individuals. These infections can result in septicemia, meningitis, endocarditis, pneumonia, wound infection, and urinary tract infections [3]. It’s the remarkable ability of *A. baumannii* to acquire resistance determinants to various antibiotics is particularly dangerous [2]. Previously, twenty-three MDR *A. baumannii* strains with the ability to form biofilms were isolated from a clinical environment in South Korea [4]. The genome of *A. baumannii* strain 1656-2, which showed particular proclivity for biofilm formation, was determined [5]. Thirteen genes, including the polyglutamic acid (PGA) synthesis related gene, were predicted for biofilm formation, and thirty-four genes related to cell adhesion were investigated in *A. baumannii* 1656-2 compared with other *A. baumannii* strains [6]. *A. baumannii* strain 1656-2 also contains more genes involved in cell motility, cell traffic, secretion, and vesicular transport than other *A. baumannii* strains [6].

Bacterial biofilm cells are highly resistant to various stresses, especially antibiotics, relative to their planktonic counterparts. This suggested that biofilm cells differ from their planktonic versions with regard to metabolic activity [7]–[9]. A global analysis of protein expression in *A. baumannii* 1656-2 has been performed [5]. The iron transporter, histidine kinase, diguanylate cyclase, antibiotic resistance, metabolism, and gene repair proteins were upregulated in biofilm environments [5].

In addition to the genome and proteomic analyses of *A. baumannii* 1656-2 strain [5], [6], we performed a global metabolite profiling of both planktonic and biofilm forms of *A. baumannii* 1656-2 using high-resolution nuclear magnetic resonance (NMR) spectroscopy. Metabolomics using nuclear magnetic resonance (NMR) spectroscopy coupled with pattern recognition analysis is useful for collecting high-density metabolite data and for characterizing metabolic changes in bacteria samples [10], [11]. Here, ^1^H NMR-spectra were collected from both biofilm and planktonic bacterial samples and subjected to multivariate statistical analyses to investigate the metabolic signature of *A. baumannii* 1656-2 biofilm cells. The present study demonstrates that NMR-based global metabolite profiling can be used to gain insight into the metabolic changes in the biofilm forming MDR *A. baumannii* 1656-2.

## Materials and Methods

### Bacterial Growth Conditions

*Acinetobacter baumannii* 1656-2 was used throughout this study. This strain was isolated from the sputum of a patient hospitalized in a university hospital in South Korea. The isolated strain exhibited the greatest ability to form biofilms among the tested strains in a previous study [4]. Cells were grown until the mid-exponential phase (OD_600_ = 0.5) in shaking flasks using M9 Minimal Media (MM) containing (per 100 ml) 2.5 ml of 20% glucose, 20 ml of x5 M9 salt (Sigma-Aldrich, St Louis, MO, USA), 200 µl of 1 M MgSO_4_, 10 µl of 1M CaCl_2_, 10 ml of 2% casamino acid, and 100 µl of trace elements. The trace elements solution contained (per 1 L) 1 g of FeSO_4_ • 7H_2_O, 1 g of MnSO_4_ • H_2_O, 0.25 g of (NH_4_)_6_Mo_7_O_24_ • 4H_2_O, 1 g of H_3_BO_3_, 0.25 g of CuCl_2_ • 2H_2_O, 0.25 g of ZnCl_2_, 0.1 g of NH_4_VO_3_, 0.25 g of Co (NO_3_)_2_ • 6H_2_O, and 2.5 g of Ca (NO_3_)_2_. The cells (OD_600_ = 0.5) were harvested to measure initial metabolites (IM) and the cultures (OD_600_ = 0.5) were diluted 50 times in MM. For static biofilm cultures, 5 ml of diluted bacterial solution was cultured in each 6 wells of a polystyrene cell culture plate (BD Falcon, Franklin lakes, NJ, USA) at 37°C without shaking and each well of 6 wells plates used as separated replicates. After 18 h static biofilm culture, unattached cells (planktonic cells, PMI) were isolated from the biofilm cells (BMI). PMII and BMII metabolites samples were generated from 72 h culture in the same manner with 18 h culture. A total of 11 samples at each stage, except for mature planktonic samples (PMII, 10 samples), were cultured to evaluate global metabolite profiling.

### Cell Extraction for Metabolite Measurements

After cultivation of the static biofilm, unattached planktonic cells were harvested in chilled tubes, pelleted by centrifugation at 5000×g for 10 min and washed twice with cold phosphate buffered saline (PBS, 137 mM NaCl, 2.7 mM KCl, 10 mM Na_2_HPO_4_, 2 mM KH_2_PO_4_, pH 7.4). Then, six well plates were washed with 5 ml of cold PBS to remove planktonic cells. After washing, the biofilm cells were detached by scrapping, pelleted in 5 ml of cold PBS by centrifugation at 5000×g for 10 min and washed with the same buffer. For intracellular metabolite extraction, cells were sonicated (sonifer 450, Branson Ultrasonics, USA) for 30 s in a mixture of methanol, chloroform, and distilled water (4∶4:2) in an ice bath, then, vortexed and left on ice for 15 min. The upper polar layer was transferred into a vial, dried using vacuum drier and stored at −80°C until NMR experiment.

### ^1^H NMR Spectroscopic Analysis

For the NMR experiment, each extract was dissolved in a 700-µL buffer solution (0.1 M phosphate buffer, pH 7.0), and pH was adjusted to 7.0±0.1. A 100 µM of sodium 3-(trimethylsilyl) propionate -2,2,3,3-d4 (TSP) in 100% D_2_O was added and a 600-µL aliquot was placed in a 5-mm NMR tube (Wilmad Lab Glass, Buena, NJ). ^1^H NMR spectra were acquired on a VNMRS-600 MHz NMR spectrometer (Agilent Technologies, Santa Clara, CA) using a triple resonance 5-mm HCN salt-tolerant cold probe. For bacterial cell extracts, the noesypresat-NOESY pulse sequence was applied to suppress the residual water signal. Free induction delays (FIDs) were collected with 16 transients into 33,784 data points using a spectral width of 8445.9 Hz with a relaxation delay of 2.0 s, an acquisition time of 4.0 s, and a mixing time of 100 ms. Signal assignment for representative samples was facilitated by acquisition of two-dimensional (2D) total correlation spectroscopy (TOCSY), correlation spectroscopy (COSY) and comparisons to the literature.

### Spectral Data Processing and Multivariate Statistical Analysis

All NMR spectra were phased and baseline-corrected using Chenomx NMR suite 6.0 (Chenomx, Edmonton, AB, Canada). The region corresponding to water (4.55–5.0 ppm) and TSP (0.00–0.70 ppm) peaks were excluded, and the remaining spectral regions were divided into 0.001 ppm bins. The binning table contained the spectral region of 0.70 to 9.50 ppm. The spectra were then normalized to the total spectral area and converted to the ASCII fotmat. The ASCII files were imported into MATLAB (R2008a; MathWorks, Inc., Natick, MA, USA), and all spectra were aligned using the correlation optimized warping (COW) method to reduce variability in the peak positions (Table S1) [12], [13]. Multivariate data analysis, principal components analysis (PCA), and orthogonal partial least-squares discriminant analysis (OPLS-DA) were performed on the binning data with mean-centered scaling using SIMCA-P (version 12; Umetrics, Umea, Sweden). PCA, an unsupervised pattern-recognition (PR) method, was used to examine the intrinsic variation in the data set, and OPLS-DA, a supervised PR method, was also employed to maximize the separation between the planktonic and biofilm cells. The quality of the models was described by *R^2^* and *Q^2^* values. *R^2^* is defined as the proportion of variance in the data explained by the models and indicates goodness of fit, and *Q^2^* is defined as the proportion of variance in the data predictable by the model and indicates predictability. In addition, we performed permutation tests and external validations to test the validity of the OPLS-DA models. Statistical significance was evaluated for individual values using the t-test for unequal variances in Excel (Microsoft, Redmond, WA, USA).

## Results

### ^1^H NMR Spectra of Planktonic and Biofilm Cells

**Figure 1:**
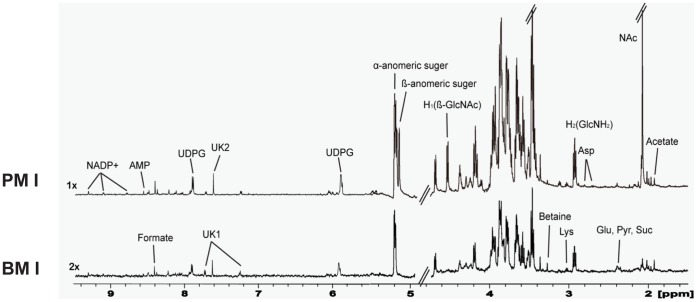
Representative 600-MHz ^1^H NMR spectra of planktonic (PMI) and biofilm (BMI) cells. UDPG, UDP-glucose; Asp, aspartate; Glu, glutamate; Pry, pyruvate; Suc, succinate; Lys, lysine; GlcNAc, *N*-acetylglucosamine; GlcNH_2_, glucosmine.

The ^1^H-NMR spectral resonances of metabolites were assigned according to the 600-MHz library from Chenomx NMR suite 6.0, 2D NMR spectra (COSY and TOCSY), and the literature (11,14,15). The concentration of metabolites was relatively higher in the early planktonic stages cells than early biofilm stages cells (**Figure 1**). In contrast, metabolite levels in the biofilm cells were higher than planktonic cells in mature stage (data not shown). Many metabolites were observed in both planktonic and biofilm cells. These include carbohydrates such as *α*-anomeric sugar, *β*-anometic sugar, glucose (Glu), H_2_(GlcNH_2_), H_1_(*β* –GlcNAc), acetate, pyruvate (Pyr) and succinate (Suc); nucleotides such as nicotinamide adenine dinucleotide phosphate (NADP^+^), UDP-glucose (UDPG) and adenosine monophosphate (AMP), amino acids such as aspartate (Asp), lysine (Lys), and glutamate (Glu), and others such as formate and betaine.

### Multivariate Statistical Analysis of Planktonic and Biofilm Cells

**Figure 2:**
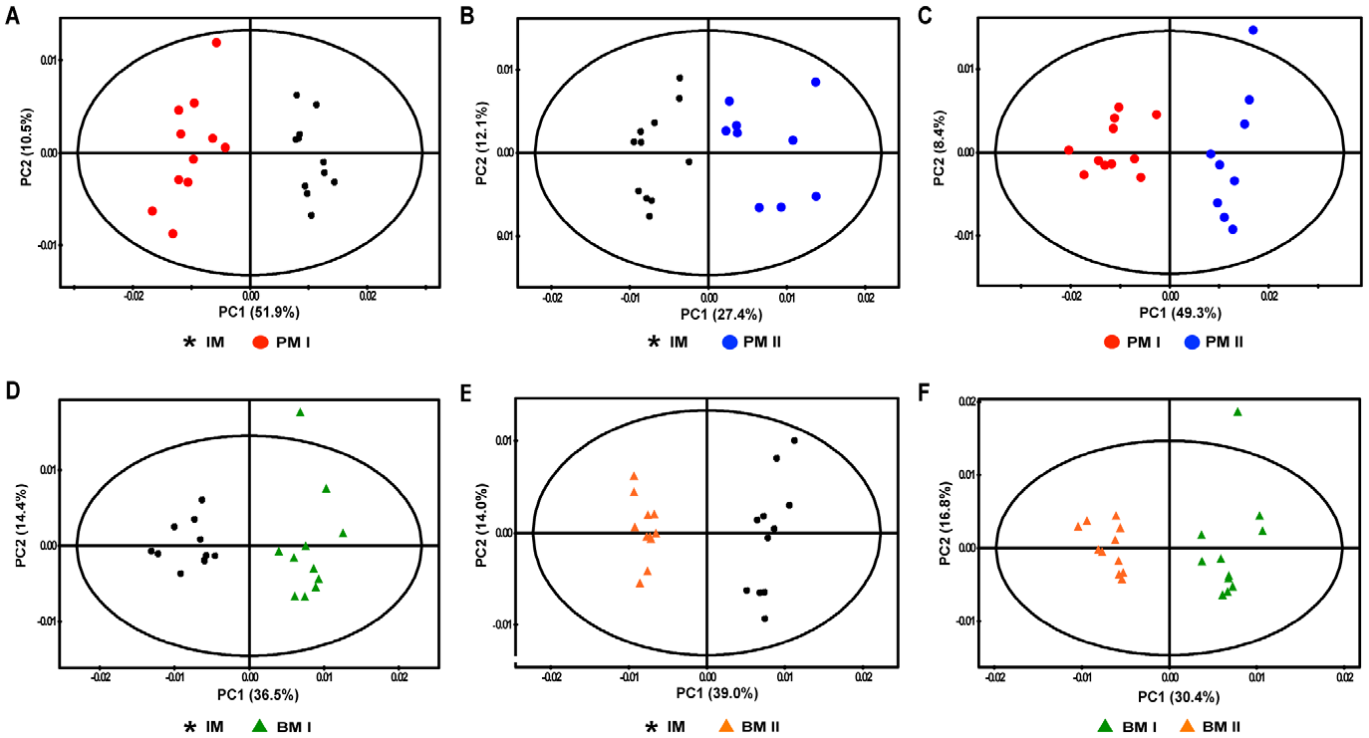
PCA score plots derived from the ^1^H NMR spectra of planktonic and biofilm cells. PCA score plots of planktonic cells demonstrated clear metabolic differences (IM/PMI, A; IM/PMII, B; PMI/PMII, C). PCA score plots of biofilm cells also indicated clear metabolic differences (IM/BMI, D; IM/BMII, E; BMI/BMII, F). Each PCA model was generated with principal components (PC). IM, initial metabolites; PMI, planktonic metabolites in the early stage; PMII, planktonic metabolites in the mature growth stage; BMI, biofilm metabolites in the early stage; BMII, biofilm metabolites in the mature growth stage.

PCA was initially performed to investigate intrinsic differences in the growth of planktonic and biofilm cells (**Figure 2**). PCA score plots of planktonic and biofilm cells revealed clear metabolic differences in the initial growth stage (IM), indicating significant changes in metabolism during the growth of *A. baumannii* 1656-2 under both planktonic and biofilm conditions (panels A, B, D, and E in **Figure 2**). Furthermore, metabolic patterns of planktonic and biofilm cells were significantly distinct (panels C and F in **Figure 2**). Therefore, *A. baumannii* 1656-2 undergoes significant changes in metabolism that are dependent on the growth state, i.e., planktonic or biofilm.

**Figure 3:**
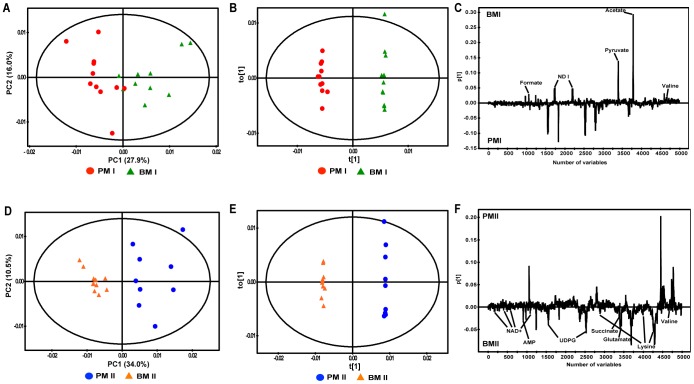
PCA and OPLS-DA score/loading plots derived from the ^1^H NMR spectra depend on growth stage. PCA and OPLS-DA score plots derived from the ^1^H NMR spectra obtained from early (A and B) and mature (D and E) stages of planktonic and biofilm cells, respectively. OPLS-DA loading plots of early (C) and mature (F) stage cells show the metabolites responsible for discrimination in the score plots. Enhanced metabolites in biofilm were reperented in the OPLS-DA loading plote. Each PCA model was generated with principal components (PC) and each OPLS-DA model was generated with predictive components (T) and orthogonal components (TO) to discriminate between groups. The PCA models for distinguishing early stage (A) and mature (D) stage cells were established using each four components.

Subsequently, the metabolic differences between planktonic and biofilm cells in the same growth stages were also examined (**Figure 3**). The resulting PCA score plots revealed significant metabolic variations with same growth stage in both planktonic and biofilm cells (panels A and D in **Figure 3**). OPLS-DA was also performed to minimize possible contributions from intergroup variability and to further improve the separation between the two groups. The score plots (panels B and E in **Figure 3**) from these OPLS-DA models for both the early (*R* ^2^X of 0.630, *R* ^2^Y of 0.997 and *Q* ^2^ of 0.839) and mature (*R* ^2^X of 0.517, *R* ^2^Y of 0.999 and *Q* ^2^ of 0.900) growth stages showed clear differentiation between planktonic and biofilm cells. The model parameters for the explained variation, *R* ^2^, and the predictive capability, *Q* ^2^, were relatively high (*R* ^2^, *Q* ^2^>0.5) in both the planktonic and biofilm models, indicating the high accuracy of the model.

The loadings plots of the bacterial cells indicate the metabolites responsible for discrimination in the score plots. The fold-changes of these metabolites were generated with their relative peak intensities from the normalized binning data of ^1^H NMR spectra. The loading plots indicate high concentrations of metabolites in the early and mature biofilm stages and several metabolites increased in the early and mature biofilm stages (**Figure 3C and F**).

To test the validity of the OPLS-DA model, we performed a permutation procedure using the PLS-DA model with the same number of components. Generally, the extrapolated intercept value of the Q^2^>0.05 indicates over-fitting in the original model. Therefore, these analyses show that our models are statistically valid (panels A and B in **Figure S1**). Other external validation processes were also performed to validate our OPLS-DA models. For the prediction, we randomly left out three beef samples from each country group and built the OPLS-DA prediction model without them. These prediction results were able to correctly predict the origins of all test beef samples in three times processes (panels C and D in **Figure S1**).

### Identification and Quantification of Metabolites from ^1^H NMR Spectra

**Figure 4:**
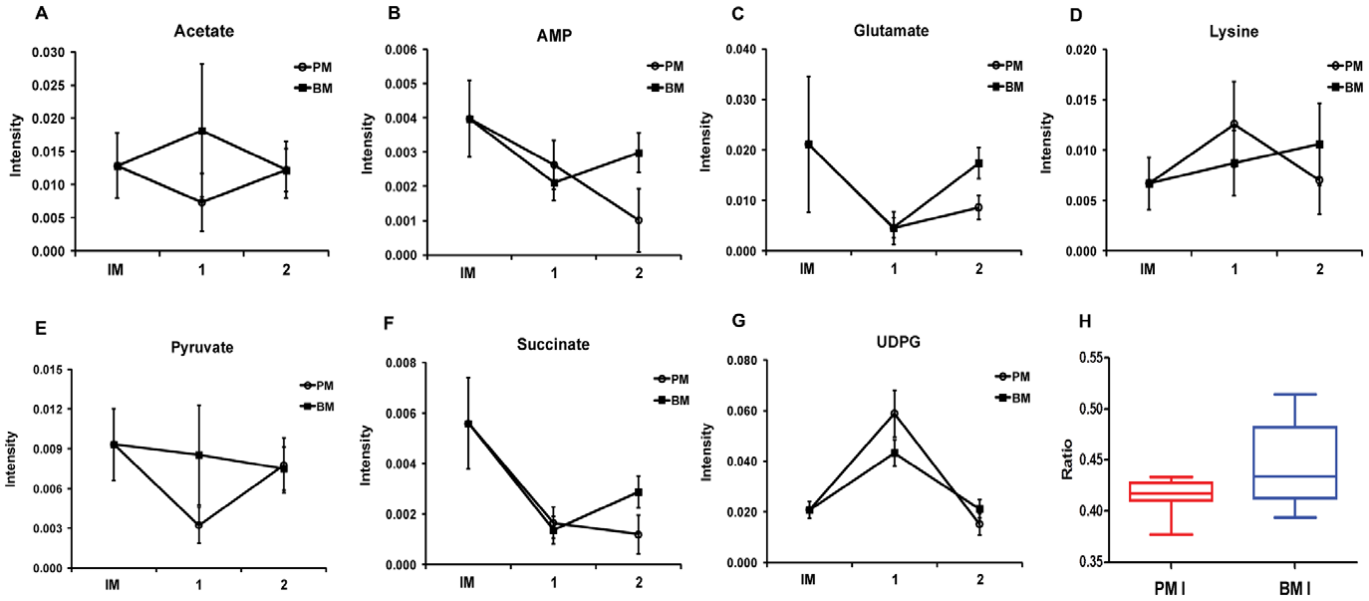
Quantification of the identified metabolites as a function growth stage in planktonic and biofilm cells. The patterns of the identified metabolites were determined as a function of growth stages (A–G). The acetylation of glucosamine was determined by a t-test (*p* = 0.026) in planktonic and biofilm cells (H).

The metabolites that were identified from NMR spectra and loading plots are listed in **Table 1**. Overall, levels of metabolites such as UDPG, acetate, AMP, glutamate, lysine, pyruvate and succinate gradually were greater in the biofilm cells than in the planktonic cells (panels A–G in **Figure 4** and **Table 1**). In the early growth stages, concentrations of several metabolites in biofilm and plank- tonic cells were similar. However, levels of metabolites in the biofilm cells increased as a function of time up to the mature growth stage. The t-test was applied to determine the significance of the differences observed between the biofilm and planktonic groups. Notably, the ratio of *N*-acetyl-D-glucosamine (GlcNAc) to D-glucosamine (GlcNH_2_) increased in the early biofilm cells compared with that of the planktonic cells (**Figure 4H** and **Figure S2**). Since understanding early phase development in biofilm formation is important [7], changes in acetyl-glucosamine level may act as markers for biofilm infections of *A. baumannii* 1656-2.

**Table 1:** Summary of the identified metabolites in planktonic and biofilm cell.

		Planktonic			Biofilm			Planktonic/Biofilm	
Metabolite	Chemicalshift	PMI/IM	PMII/IM	PMI/PMII	BMI/IM	BMII/IM	BMI/BMII	PMI/BMI	PMII/BMII
Acetate	1.91	0.57	0.95	0.60	1.41	0.95	1.48	0.41	1.00
AMP	8.60	0.66	0.26	2.59	0.53	0.75	0.71	1.25	0.34
Aspartate	2.82/2.79	2.95/6.09	4.22/5.75	0.70/1.06	1.87/3.13	1.59/4.77	1.17/0.66	1.58/1.95	2.65/1.21
Betaine	3.25	0.42	1.05	0.40	0.40	0.66	0.61	1.05	1.57
Formate	8.44	0.40	1.01	0.40	0.47	0.50	0.95	0.85	2.04
Glutamate	2.33	0.21	0.41	0.52	0.22	0.82	0.26	0.98	0.49
Lysine	3.01/1.72	2.36/1.88	1.61/1.05	1.47/1.79	1.60/1.30	1.77/1.58	0.90/0.82	1.48/1.44	0.91/0.66
NAD+	9.34	1.52	0.26	5.81	1.16	0.97	1.20	1.31	0.27
Pyruvate	2.38	0.35	0.83	0.42	0.91	0.81	1.14	0.38	1.03
Succinate	2.39	0.30	0.21	1.39	0.24	0.52	0.47	1.21	0.41
UDPG	7.94	2.83	0.73	3.88	2.08	1.02	2.04	1.36	0.71
Valine	1.03	0.22	0.33	0.65	0.61	0.38	1.62	0.35	0.88
ND I	7.76/7.30	0.29/0.27	0.70/0.75	0.42/0.36	0.84/0.77	0.38/0.31	2.24/2.47	0.35/0.35	1.86/2.40
ND II	7.67	1.20	1.91	0.63	0.54	1.71	0.31	2.24	1.12
ND III	3.10/2.11	1.26/2.23	1.33/0.83	0.94/2.68	0.50/1.62	0.48/1.14	1.04/1.42	2.51/1.38	2.77/0.73

a ND: not determined.

## Discussion

Control of MDR bacteria is important for public health [16]. MDR *A. baumannii* strains are becoming more common in hospitals and worldwide, raising the need for effective therapies [2]. The formation of a biofilm enhances the antibiotic resistance of bacteria and contributes to the chronicity of infections such as those associated with implanted medical devices in hospital [7]. However, the mechanisms of resistance in biofilms differ from the familiar mechanisms of innate resistance [8]. Therefore, understanding the physiological response of biofilms is important for developing effective treatments. While global metabolite analysis has been shown as a powerful tool for understanding bacterial physiology, it had not been applied to in MDR *A. baumannii* biofilm cells. The current study describes the NMR-based global metabolic profiling of MDR *A. baumannii* 1656-2 stain under both biofilm and planktonic conditions. This provides an excellent means of understanding biofilm formation in *A. baumannii*1656-2.

### Carbohydrate Metabolism

The metabolic profiles in **Table 1** and **Figure 4** show that levels of succinate, pyruvate and acetate, which are related to energy metabolism, were higher in the mature biofilm than in the planktonic growth stage, indicating a reduction in energy production. This is consistent with a previous report that showed a slower growth rate for biofilms relative to the growth rate of the same bacteria in its planktonic form. The biofilm also allows the bacteria to survive under unfavorable conditions [17], [18]. Experiments with *Pseudomonas aeruginosa* have shown that the deeper layer in a biofilm are anaerobic or microaerobic and these deeper layers were supported by-pyruvate fermentation [19]. As confirmation, metabolites such as succinate, lactate, and acetate, which are final products of pyruvate fermentation, were detected in *P. aeruginosa* biofimls by HPLC analysis [19]. Notably, the current study yielded high levels of acetate, pyruvate, and succinate in the mature biofilm (**Table 1** and **Figure 4**). Therefore, cells in an *A. baumannii* 1656-2 biofilm may depend on pyruvate fermentation for long-term survival. Also, biofilm cells have been proposed to accumulate acetate, which facilitates the formation of biofilms in *Escherichia coli* [20]. In this study, levels of acetate were higher in biofilm samples than in planktonic bacteria. Thus, acetate may be important to the formation of *A. baumannii* 1656-2 biofilm (**Table 1** and **Figure 4**). Changes in the carbohydrate metabolism may allow *A. baumannii* 1656-2 biofilm cells to grow with little or no oxygen by making use of pyruvate fermentation.

### Nucleotide Metabolism

Levels of UDPG, AMP and NAD^+^ were higher in the mature biofilm than planktonic stage (**Table 1** and **Figure 4**). UDPG can be used as a precursor of polysaccharides such as exopolysaccharide (EPS), which are important to maintaining biofilms of *Staphylococcus epidermidis, Vibrio parahaemolyticus,* and *A. baumannii* [9], [21], [22]. In particular, proteome data on *A. baumannii* biofilm cells indicated that EPS matrix formation occurs via the Leloir pathway [23]. Moreover, UDPG level in biofilm cells increased in this study (**Table 1** and **Figure 4**). The Leloir pathway comprises a step in galactose phosphorylation (galactose-^1^P). EPS production in the cell requires free P_i_ and Leloir pathway acts as the P_i_-supplier for biofilm formation [23]. Levels of AMP were also higher in the mature biofilm, which suggests that *A. baumannii* 1656-2 biofilm cells require a relatively large supply of P_i_ for biofilm formation. In *A. baumannii*, histidine degradation leads to nucleotide production [23]. Since extracellular DNA (eDNA) is an important constituent of the biofilm matrix [9], histidine degradation may contribute to nucleotide synthesis for eDNA production. High levels of AMP in the mature biofilm may help to maintain biofilm structure (**Table 1** and **Figure 4**). *Thermotoga maritima* biofilm cells exhibited increased transcription of genes involved in the biosynthesis of NAD^+^ for iron-sulfur cluster biogenesis [15]. Furthermore, induction of oxidative stress defense protein, which uses NAD^+^ as a cofactor, has been observed in biofilm cells [24]. This is consistent with the higher levels of NAD^+^ observed in the mature biofilm (**Table 1**). An association between oxidative stress and biofilm formation has been observed in bacterial systems [15]. Therefore, oxidative stress may promote biofilm formation in *A. baumannii* 1656-2.

### Amino Acid Metabolism

Levels of valine, glutamate, and lysine were enhanced in the mature *A. baumannii* 1656-2 biofilm (**Table 1** and **Figure 4** **)**. Valine was previously shown to be continuously secreted in bacterial biofilm [25]. This is consistent with the present data, which shows steady valine production in the biofilm cells (**Table 1** **)**. Valine accumulates in biofilm cells, because of reduced-diffusion and the high-cell-density of the biofilm matrix [25]. Valine synthesis likely contributes to biofilm matrix maintenance, and glutamate metabolism has been reported to be essential for biofilm formation [23]. The present results are consistent with this observation, as glutamate levels increased in the mature biofilm (**Table 1** and **Figure 4** **)**. Lysine, which was also observed in the mature *A. baumannii* 1656-2 biofilm (**Table 1** and **Figure 4**), was shown to be important for biofilm accumulation and the epithelial barrier to bacterial proinflammatory agents [26]. Amino acid levels increased in biofilm cells. Amino acids are used as precursors for energy production with gluconeogenesis [27]. A high demand for amino acids as substrates for energy production may exist in harsh environments such as those encountered in a biofilm [7].

### Effect of *N*-Acetyl-D-glucosamine on Biofilm Formation

Production of an extracellular mixture of sugar polymers called an EPS is critical for biofilm formation [21], [28]. In particular, *N*-acetyl-D-glucosamine (GlcNAc) appears to influence a variety of cellular processes, including energy metabolism, chitin utilization, competence, biofilm formation and pathogenicity in bacteria [22]. For example, a homopolymer of GlcNAc is located on the cell surfaces of *Staphylococcus epidermidis* and *S. aureus*, where it serves as an essential factor for biofilm formation [21], [29]. Only 60% of the glucosamine amino groups are acetylated and GlcNAc is useful vaccine candidate against MDR *A. baumannii* [30]. The data herein shows a high ratio of GlcNAc to GlcNH_2_ in the early biofilm stage (**Figure 4H**). Thus, high levels of acetyl-glucosamine may be used as a marker for biofilm formation with MDR *A. baumannii*. Note that a small percentage of the GlcNAc residues in each polysaccharide were deacetylated [31], [32]. Thus, the ratio of GlcNAc to GlcNH_2_ may be lower in mature biofilm structures.

In this study, planktonic and biofilm cells were harvested at low temperature condition. It has been reported that various quenching procedures can be used to quickly arrest metabolism [33]. In particular, rapid sampling and reliable quenching techniques are very important in bacterial cells because of the metabolite leakage. Therefore, the results of this study can be weakened by the prolonged sampling procedures. PBS washing procedure was commonly used for analysis of biofilm cells [34]. It has been reported that the precooled PBS (pH 7.4, 0.5°C) is the optimal quenching reagent for fixing intracellular metabolism [35]. Although our data have a vulnerable point about quenching procedure, this study demonstrates that the analysis of intracellular metabolites in biofilm cells using ^1^H NMR based metabolomics is useful for understanding biofilm formation in MDR bacteria.

## Supporting Information

Figure S1 **The permutation test of PLS-DA models and the external validation test of OPLS-DA models.** The permutation tests of PLS-DA model were performed by BMI/PMI (A) and BMII/PMII (B) with 100. Samples of BMI/PMI (C) and BMII/PMII (D) were analyzed by external validation test.
(DOC)

Figure S2 **An expansion of 2D TOCSY 1H NMR spectrum of early planktonic stage cell.** Representative result of 2D TOCSY 1H NMR spectrum.
(DOC)

Table S1 **The binning table of 0.70 to 9.50 ppm region.** The region corresponding to water and TSP regions were excluded, and the remaining spectral regions were divided into 0.001 ppm bins.
(XLS)
