# Supplementary material for: 1H NMR-Based Metabolite Profiling of Planktonic and Biofilm Cells in Acinetobacter baumannii 1656-2
Source: PLoS One. 2013 Mar 6;8(3):e57730. doi: 10.1371/journal.pone.0057730 (PMC3590295; doi:10.1371/journal.pone.0057730)
Supplement: Figure S1 — The permutation test of PLS-DA models and the external validation test of OPLS-DA models. The permutation tests of PLS-DA model were performed by BMI/PMI (A) and BMII/PMII (B) with 100. Samples of BMI/PMI (C) and BMII/PMII (D) were analyzed by external validation test. (DOC) [file pone.0057730.s001.doc]

**Figure S1**


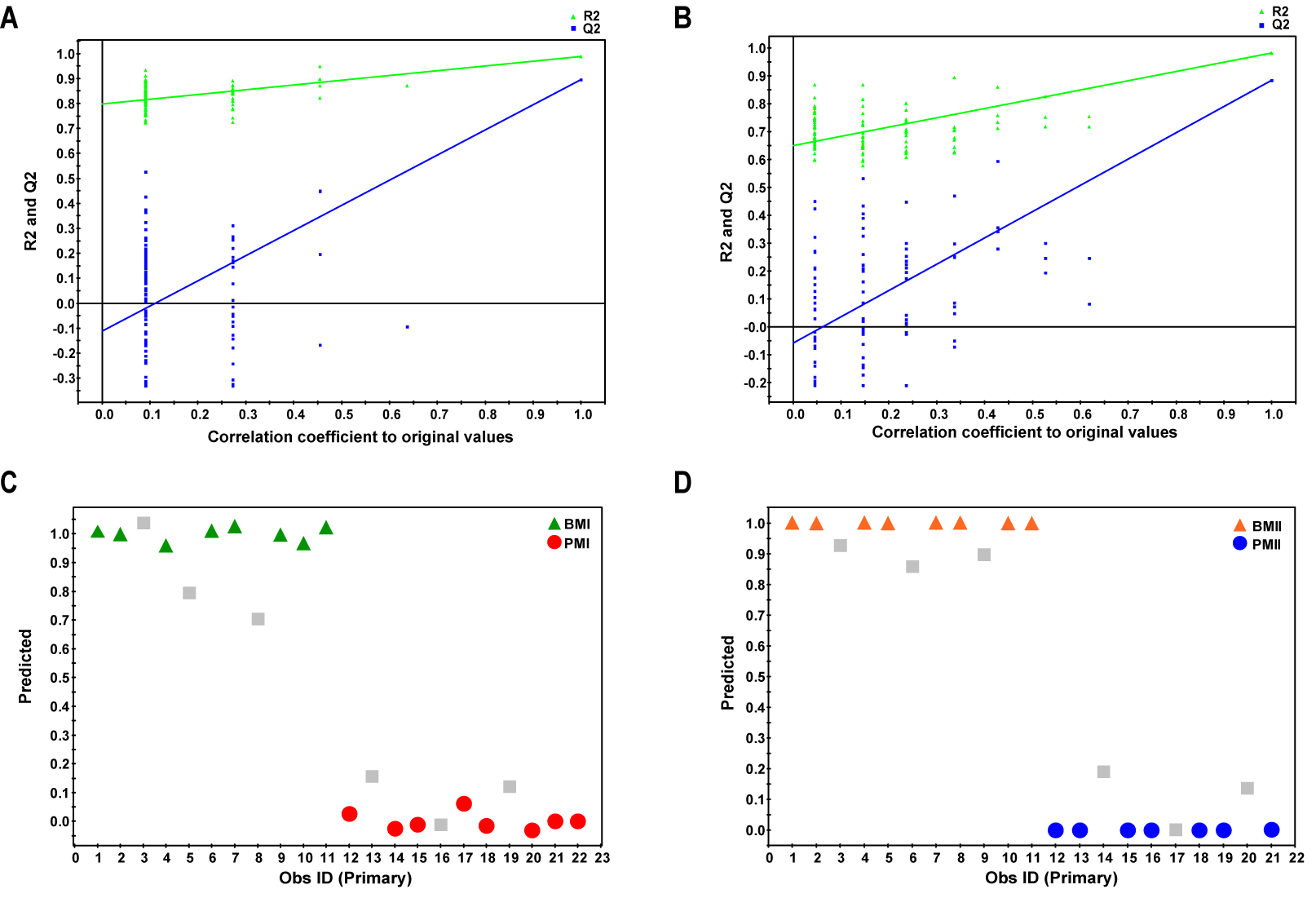


**Supplementary Figure S1.** The permutation test of PLS-DA models: The permutation tests of PLS-DA model were performed by BMI/PMI (A) and BMII/PMII (B) with 100 permutations. The external validation test of OPLS-DA models: Samples of BMI/PMI (C) and BMII/PMII (D) were analyzed by external validation test. The processes were performed three times by leaving randomly three samples (a test data set) and constructing new models with only the remaining eight samples (a training data set), except for PMII sample (seven samples). Test samples can be considered unknown samples and are shown as gray squares.
