# Supplementary material for: 1H NMR-Based Metabolite Profiling of Planktonic and Biofilm Cells in Acinetobacter baumannii 1656-2
Source: PLoS One. 2013 Mar 6;8(3):e57730. doi: 10.1371/journal.pone.0057730 (PMC3590295; doi:10.1371/journal.pone.0057730)
Supplement: Figure S2 — An expansion of 2D TOCSY 1H NMR spectrum of early planktonic stage cell. Representative result of 2D TOCSY 1H NMR spectrum. (DOC) [file pone.0057730.s002.doc]

**Figure S2**


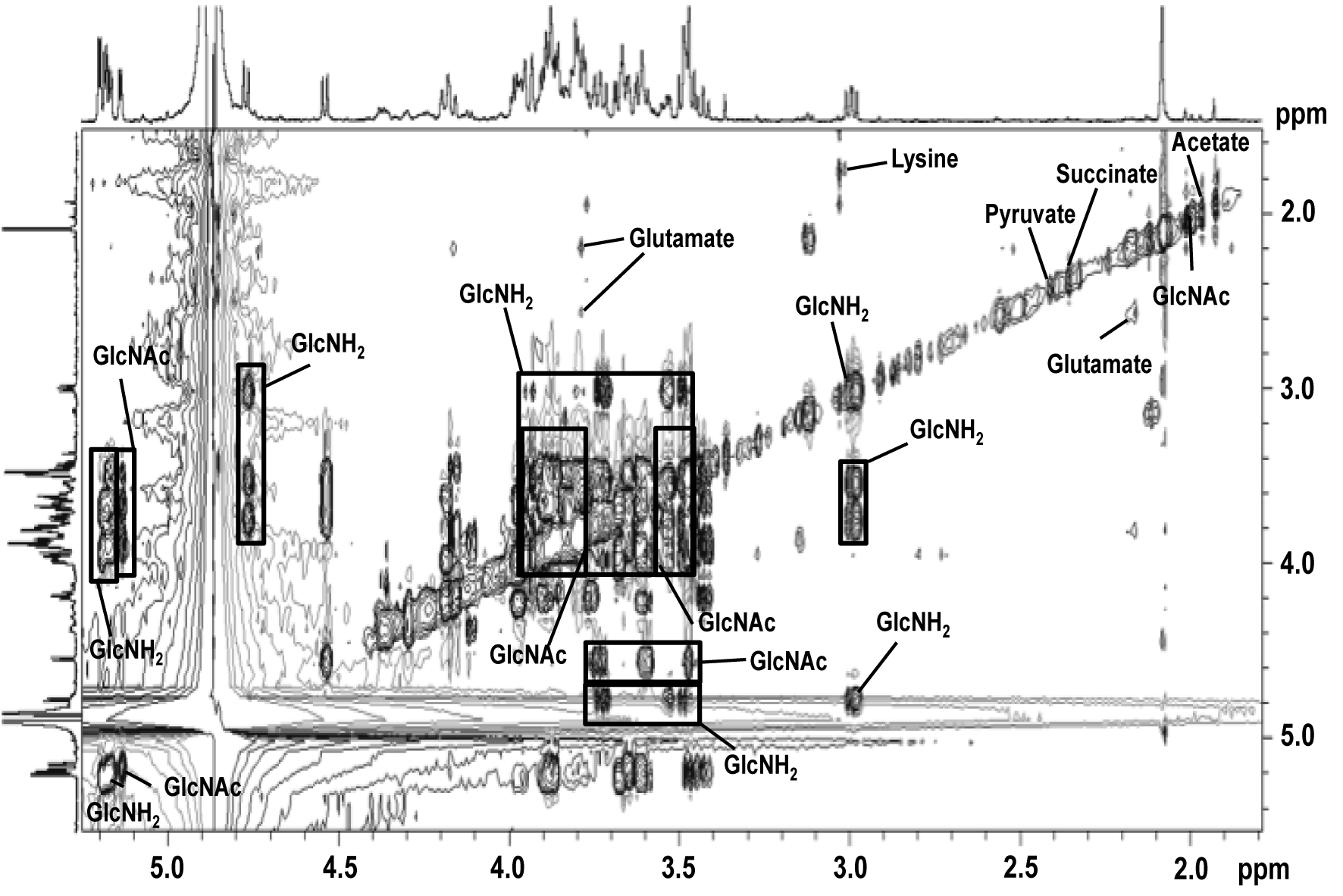


**Supplementary Figure S2.** An expansion of 2D TOCSY ^1^H NMR spectrum of early planktonic stage cell.
